# Identification of a genetic risk factor for metformin-induced vitamin B_12_ deficiency

**DOI:** 10.1007/s00125-025-06655-5

**Published:** 2026-01-15

**Authors:** Faye D. Baldwin, Khaled F. Bedair, Andrea L. Jorgensen, Lewis Green, Innocent G. Asiimwe, Colin N. A. Palmer, Archie Campbell, Caroline Hayward, Qing Pan, Shiyu Shu, Josephine H. Li, Diabetes Prevention Program (DPP) Research Group, Ewan R. Pearson, Munir Pirmohamed, Daniel F. Carr

**Affiliations:** 1 Department of Health Data Science, University of Liverpool, Liverpool, UK; 2 Division of Population Health & Genomics, School of Medicine, Ninewells Hospital & Medical School, University of Dundee, Dundee, UK; 3 Department of Pharmacology and Therapeutics, University of Liverpool, Liverpool, UK; 4 Centre for Genomics and Experimental Medicine, Institute of Genetics and Cancer, University of Edinburgh, Edinburgh, UK; 5 Medical Research Council Human Genetics Unit, Medical Research Council Institute of Genetics and Cancer, University of Edinburgh, Edinburgh, UK; 6 Department of Epidemiology and Biostatistics, George Washington University Biostatistics Canter, Washington, DC USA; 7 Center for Genomic Medicine, Massachusetts General Hospital, Boston, MA USA; 8 Diabetes Unit, Department of Medicine, Massachusetts General Hospital, Boston, MA USA; 9 Programs in Metabolism and Medical & Population Genetics, Broad Institute of Harvard and MIT, Cambridge, MA USA; 10 Harvard Medical School, Boston, MA USA

**Keywords:** Cobalamin deficiency, Genome-wide association study, Metformin, Type 2 diabetes, Vitamin B_12_ deficiency

## Abstract

**Aims/hypothesis:**

Metformin, a mainstay of treatment for type 2 diabetes, can cause vitamin B_12_ deficiency. Clinical risk factors have been identified but genetic factors remain undiscovered. Our objective was to identify and validate genetic predisposing factors and establish clinical utility.

**Methods:**

Individuals with metformin-induced vitamin B_12_ deficiency (*n*=487) and metformin-tolerant control individuals (*n*=6686) were identified in UK Biobank. Genome-wide association analysis was undertaken using logistic regression. Replication was undertaken in three cohorts: a Scottish cohort; the Diabetes Prevention Program Outcomes Study (DPPOS); and a separate cohort from Liverpool. In the Liverpool cohort, plasma metformin levels were also measured.

**Results:**

Analysis identified a genome-wide significant non-synonymous SNP in the cubilin gene (*CUBN,* rs1801222/p.S253F) associated with metformin-induced vitamin B_12_ deficiency (additive model; adjusted *p*=1.86×10^−10^; OR 1.56 [95% CI 1.36, 1.79] for AG vs GG genotype; OR 2.43 [95% CI 1.85, 3.20] for AA vs GG genotype), which was replicated in both the Scottish and the DPPOS cohorts. Vitamin B_12_ deficiency occurred in 0.84–1.20% of non-metformin-exposed individuals regardless of rs1801222 genotype. However, a large interaction with metformin use was observed, with vitamin B_12_ deficiency occurring at 6.02% in GG, 7.96% in GA and 12.84% in AA genotype groups. When followed up from metformin initiation, 10% with the AA genotype were vitamin B_12_ deficient by 11 years vs 21 years for 10% of the GG group.

**Conclusions/interpretation:**

The observed genetic association suggests that the rs180122 genotype should be considered a significant risk factor for metformin-induced vitamin B_12_ deficiency. While clinical monitoring of serum vitamin B_12_ levels in patients on metformin is inconsistently done, this finding highlights the potential clinical utility of targeted monitoring for certain subsets of individuals, including those genetically at high risk.

**Graphical Abstract:**

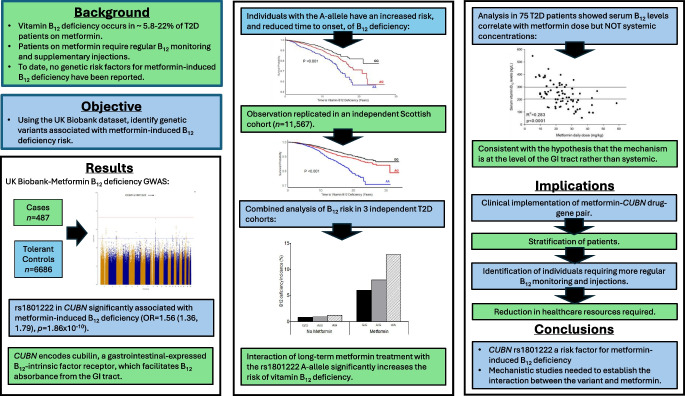

**Supplementary Information:**

The online version contains peer-reviewed but unedited supplementary material available at 10.1007/s00125-025-06655-5.


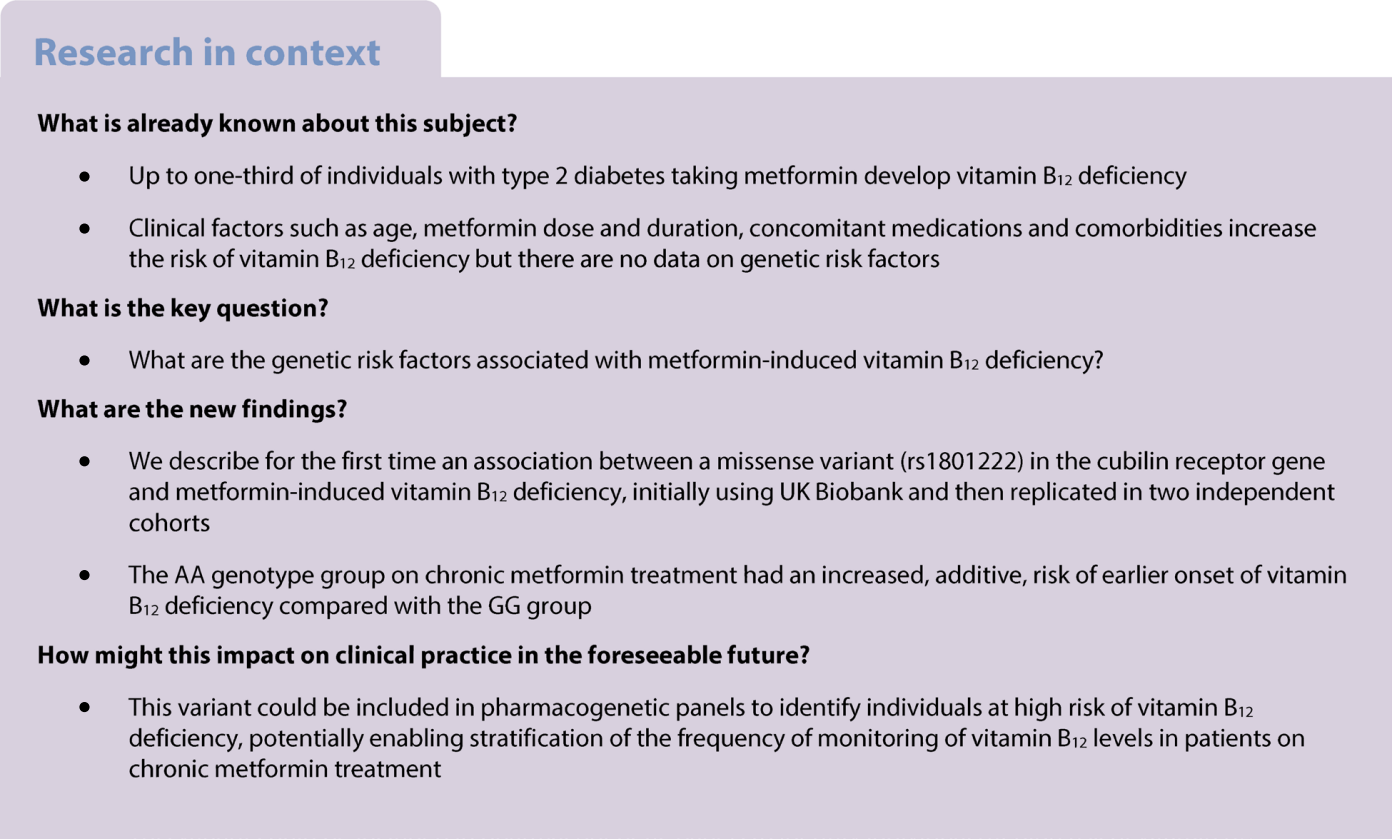


## Introduction

Metformin, the primary treatment for type 2 diabetes, is largely well tolerated but can cause vitamin B_12_ deficiency in 5.8–22% [1, 2] of patients. The Diabetes Prevention Program Outcomes Study (DDPOS) showed that combined low and borderline-low vitamin B_12_ levels were more common in metformin users after 5 and 13 years of use, compared with levels in non-users. Each year of metformin use increased the risk of vitamin B_12_ deficiency by about 13% [3].

Intestinal malabsorption has been implicated in vitamin B_12_ deficiency in metformin users, although the exact mechanism is unclear. Some investigators have argued that biochemical vitamin B_12_ deficiency does not represent true deficiency but merely reflects altered vitamin B_12_ tissue distribution and metabolism [4, 5]. However, the demonstration of increased homocysteine levels and the occurrence of anaemia and neuropathy [3] in metformin users suggests that biochemical vitamin B_12_ deficiency may be clinically relevant [6]. Furthermore, a systematic review of 43 studies showed that vitamin B_12_ levels in the subclinical low-normal range (<250 ng/l) were associated with Alzheimer's disease, vascular dementia and Parkinson's disease, while vitamin B_12_ deficiency (<150 ng/l) was associated with cognitive impairment [7].

Clinical risk factors for metformin-induced vitamin B_12_ deficiency include higher metformin dose, longer treatment duration [8], older age, low baseline vitamin B_12_ levels, low dietary vitamin B_12_ intake and concomitant medications (e.g. proton pump inhibitors [PPIs] [8]). The Medicines and Healthcare products Regulatory Agency (MHRA), the UK’s medicines regulator, has highlighted the importance of checking vitamin B_12_ levels in patients on metformin, particularly in those with risk factors, and correcting any deficiency [9]. Unfortunately, regular monitoring for vitamin B_12_ deficiency in metformin users is uncommon [10, 11].

Although a number of studies have reported genetic associations with serum vitamin B_12_ levels [12–14] or generalised vitamin B_12_ deficiency [15], to date no genetic variants predisposing to metformin-induced vitamin B_12_ deficiency have been identified. Therefore, our objective was to identify genetic predisposing factors associated with metformin-induced vitamin B_12_ deficiency. We utilised the UK Biobank (UKB) to identify individuals on metformin with and without vitamin B_12_ deficiency and performed a genome-wide association study (GWAS) to identify associated genetic variants, with replication in independent cohorts.

## Methods

### Phenotype definitions

#### Vitamin B_12_ deficiency

Individuals with a record of vitamin B_12_ deficiency were identified from ICD9 and ICD10 Read v2 and v3 codes (electronic supplementary material [ESM] Table 1) and/or a record of B_12_ injection prescriptions (ESM Table 2).

#### Metformin users with vitamin B_12_ deficiency

Individuals with a type 2 diabetes diagnosis recorded in primary care/hospital records and ≥1 metformin prescription with subsequent vitamin B_12_ deficiency diagnosis or vitamin B_12_ injection prescription post-first metformin prescription were identified. Index date was set as the first recorded vitamin B_12_ deficiency or prescription of vitamin B_12_ injection, whichever was earliest.

#### Metformin-tolerant control individuals

Individuals with a type 2 diabetes diagnosis and ≥1 metformin prescription and no subsequent vitamin B_12_ deficiency diagnosis or vitamin B_12_ injection were identified. The index date applied to tolerant control individuals, for assessment of concomitant medications, was set as mean time from metformin initiation to onset of vitamin B_12_ deficiency/injection observed in the vitamin B_12_-deficient metformin users (6.88 years).

### Identification of cases and controls in UKB

Metformin vitamin B_12_-deficient cases and controls were identified from the ~500,000 participants in the UKB cohort enrolled from 2006–2010, aged 40–69 and followed up to September 2017 [16]. Clinical codes (https://biobank.ndph.ox.ac.uk/ukb/refer.cgi?id=592) contain all the British National Formulary (BNF), National Health Service (NHS) Dictionary of Medicines and Devices (DMD) and Read drug codes and brand names. The full code list used for prescription and diagnosis are available in ESM Tables 1–4. Individuals with type 2 diabetes were identified from UKB data fields 130708 and 130709. This process included information from the death register, primary care data, hospital admissions data and self-reported data.

Individuals with one or more metformin prescription were identified in GP prescription records (UKB data field 42039) based on drug name (including brand names), Read v2, DMD and BNF codes. Similarly, this approach identified individuals with one or more prescription for injectable vitamin B_12_.

Individuals diagnosed with vitamin B_12_ deficiency or pernicious anaemia were identified from GP clinical event records (UKB data field 42040) and by mapping ICD9, ICD10 and Read v2 and v3 codes related to vitamin B_12_ deficiency. Those with a history of vitamin B_12_ deficiency, vitamin B_12_ injection or pernicious anaemia prior to commencing metformin administration, and for whom cumulative metformin dose could not be calculated (missing dose data), were excluded.

### Covariates

The methodology for obtaining covariate data from UKB and details of covariate selection and rationale can be found in the ESM Methods (Covariate selection). Gender was initially obtained from self-reported data and subsequently confirmed from the genotyping data.

### Genotyping and imputation

Genotyping and imputation processes for the UKB cohort, including sample and SNP quality control (QC) procedures, have been previously reported [17] and are summarised in ESM Methods under UKB genotyping and Per-participant quality control.

### Primary analysis

Genome-wide logistic regression analysis of cases vs controls was undertaken, assuming an additive mode of inheritance, using SNPTEST version 2.5.6 [18] (primary outcome: risk of metformin-induced vitamin B_12_ deficiency). The following covariates were adjusted for: gender; age at first metformin prescription; cumulative dose; duration of metformin use; use of PPIs 6 months prior to index date (Table 1); plus the first ten principal components accounting for genetic ancestry among White European individuals as calculated by UKB [17]. Two significance thresholds were used, including a Bonferroni multiple testing-corrected genome-wide statistical significance threshold of *p*<5×10^−8^ to control for false-positives and a suggestive significance threshold of *p*<1×10^−5^. Manhattan and quantile–quantile plots were created using R package qqman (v0.1.9) [19].

**Table 1:** UKB cohort demographics and clinical characteristics included in the primary analysis of UKB data

Variable	Cases (*n*=487)	Controls (*n*=6686)	OR (95% CI)	*p* value
Female gender, *n* (%)	205 (42)	2487 (37)	1.25 (1.03, 1.52)	0.020
Age at first metformin prescription, years	59±8	61±8	1.01 (1.00, 1.03)	0.012
Time to vitamin B_12_ event, years	6.88±4.77	NA	-	-
Cumulative metformin dose, mg	6.00×10^6^±4.33×10^6^	3.84×10^6^±3.03×10^7^	1.00 (n/a)	0.64
Duration of metformin use, years	9.0±5.2	6.2±4.9	1.09 (1.06, 1.11)	<1.64×10^−14^
PPI use (6 months prior), *n* (%)	216 (44)	1046 (16)	3.42 (2.80, 4.16)	<2×10^−16^

Data are presented as mean ± SD or *n* (%)

*p* value and OR (95%CI) are determined by multivariate logistic regression analysis

Cases, metformin-associated vitamin B_12_ deficiency; controls, metformin tolerant

For SNPs with *p*<5×10^−8^, a comparable logistic regression analysis of metformin-induced vitamin B_12_ deficiency was undertaken in a cohort limited to self-reported Pakistani, Bangladeshi or Indian ancestry (South Asian) identified in the UKB cohort, adjusting for the same covariates, to determine if the association observed was applicable to anther ancestry group.

### Secondary analyses

First, to explore whether observed genetic associations were specific to metformin-induced vitamin B_12_ deficiency, rather than generalised vitamin B_12_ deficiency, a secondary analysis was undertaken for SNPs meeting genome-wide significance in the primary analysis. Cases were defined as individuals with type 2 diabetes and metformin-induced vitamin B_12_ deficiency as defined above while controls were defined as individuals with type 2 diabetes with no history of metformin use but with a vitamin B_12_ deficiency diagnosis or history of receiving a vitamin B_12_ injection (index date was the first report of vitamin B_12_ deficiency). A logistic regression model of cases vs controls was applied assuming an additive mode of inheritance, adjusting for the first ten principal components of genetic ancestry, gender and prior use (6 months) of PPIs.

Second, further analysis compared cases, defined as individuals with a history of vitamin B_12_ deficiency/B_12_ injections vs controls (individuals with no history of vitamin B_12_ deficiency or vitamin B_12_ injections, irrespective of having previously been treated with metformin). This was undertaken for SNPs meeting genome-wide significance in the primary analysis. A logistic regression model adjusted for gender and the first ten principal components of genetic ancestry was fitted but with a binary metformin covariate (treated with/not treated with metformin), plus a SNP × metformin interaction covariate, with additive mode of inheritance assumed.

### Survival analysis

Survival analysis of statistically significant SNPs (*p*<5×10^−8^) from the primary analysis was undertaken, comparing time to metformin-induced vitamin B_12_ deficiency (primary outcome) stratified by genotype. Outcome was time to first vitamin B_12_ deficiency event (either clinical vitamin B_12_ deficiency diagnosis or receipt of vitamin B_12_ injection), which was calculated as the number of days from first metformin prescription to the first vitamin B_12_ deficiency event. For individuals who did not experience the primary outcome, censoring was applied on the last recorded metformin prescription date. Both unadjusted and adjusted Cox regression models were fitted, with the adjusted Cox regression models using the same variables as in the primary logistic regression analysis. Kaplan–Meier plots were prepared, stratified by genotype.

### Replication cohorts

#### Scottish cohort

Data up to April 2022 from three cohorts within the Tayside Bioresource, University of Dundee was utilised:

- Generation Scotland (GS): the Scottish Family Health Study is a family-based genetic epidemiology study conducted from February 2006 to March 2011. It includes DNA, sociodemographic and clinical data from ~24,000 volunteers across Scotland, aged 18–98 years.
- GoDARTS: includes 18,306 participants aged 16–98 years, 10,149 with type 2 diabetes and 8157 without. Baseline data are available for 16,838 participants (8698 cases and 8140 controls), with genetic data available for 8564 type 2 diabetes cases and 4586 controls.
- SHARE: The Scottish Health Research Register (SHARE) (established in 2011) consists of ~130,000 participants.

Additional information can be found in ESM Methods under Scottish Replication Cohort. Further details on the Tayside, Scotland cohorts, can also be found in [20–23].

Individuals with one or more prescriptions for metformin were identified from GP prescription records, using approved drug names (ESM Table 3). Codes for injectable vitamin B_12_ preparations are noted in ESM Table 4.

#### Diabetes Prevention Program cohort

The Diabetes Prevention Program (DPP) and its long-term follow-up study, DPPOS, was a multicentre RCT that evaluated whether intensive lifestyle modification or metformin 850 mg twice daily, compared with placebo, prevented or delayed type 2 diabetes in high-risk individuals [24]. The study population comprised participants randomised to placebo (*n*=902) or metformin (*n*=898), with available serum vitamin B_12_ levels from DPPOS years 1 or 9 (~5 and 13 years after randomisation). Genotype data for rs1801222 was available for 1417 and 1221 participants in DPPOS years 1 and 9, respectively.

#### Liverpool replication cohort

Seventy-five patients (45 male/30 female; mean age 64 years; range 42–80 years) receiving long-term metformin treatment for type 2 diabetes (mean duration 10.17 years; range 0.3–31.67 years) were recruited between September 2007 and October 2009. Ethical approval was obtained from the Liverpool Ethics Research Committee. All individuals gave informed written consent before participating. Details of DNA extraction, genotyping, plasma metformin and serum vitamin B_12_ measurements are detailed in ESM Methods (Liverpool Replication Cohort) and ESM Table 5.

### Meta-analysis

Data from all cohorts were combined in meta-analyses, one each for comparing AG to GG and AA to GG genotypes, in both metformin users and non-users, using the Mantel–Haenszel random-effects approach to obtain pooled estimates. For the UKB cohort, data on vitamin B_12_ deficiency in metformin non-users were obtained from the data used in the second Secondary Analysis

## Results

### Primary analysis of UKB cohort

A total of 47,023 individuals diagnosed with type 2 diabetes were identified in UKB; 42,400 had type 2 diabetes confirmed in UKB primary care data. Of these, 11,042 had one or more prescription for metformin. After applying exclusion criteria and genotype QC, a total of 487 metformin-induced vitamin B_12_-deficient cases and 6686 metformin-tolerant controls were identified (Table 1 and ESM Fig. 1). A total of 8,669,584 SNPs were analysed, having passed per-variant QC thresholds.

**Fig. 1:**
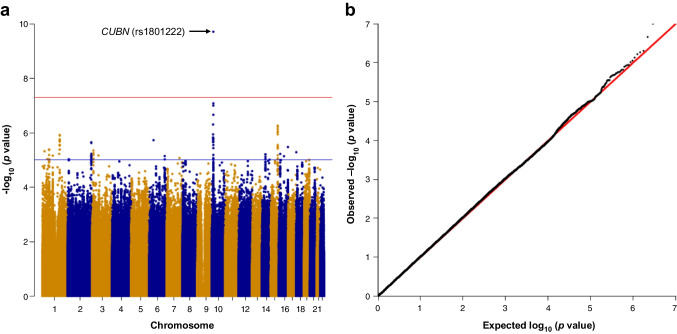
Manhattan plot of primary analysis of UKB data. (**a**) The *y*-axis represents –log_10_ *p* value for logistic regression analysis, assuming an additive mode of inheritance, and the *x*-axis indicates the chromosomal position of each SNP. (**b**) Quantile–quantile plot of the genome-wide association analysis of metformin-induced vitamin B_12_ deficiency. The *y*-axis represents –log_10_ *p* value for logistic regression analysis and the *x*-axis indicates –log_10_ of the expected *p* values given the number of markers. Red line indicates genome-wide statistical significance (5×10^-8^). Blue line indicates nominal statistical significance (1×10^-5^)

Genome-wide association analysis (Fig. 1 and ESM Table 6) identified one non-synonymous SNP in the *CUBN* gene (encoding cubilin) (rs1801222, XP_011518010.1:p.S253F, NM_001081.4: c.758T>C), which passed the genome-wide significance threshold (additive model; adjusted *p*=1.86×10^−10^; unadjusted *p*=5.99×10^−11^ ; OR 1.56 [95% CI 1.36, 1.79] for AG vs GG; OR 2.43 [95% CI 1.85, 3.20] for AA vs GG). A further 877 SNPs reached suggestive statistical significance (*p*<1×10^−5^). The top ten associated loci are summarised in ESM Table 6 and were analysed using both the GWAS catalogue [25] and Gene Atlas [26] for previously reported associations with vitamin B_12_ deficiency, or related traits or phenotypes. Only rs180122 (risk allele=A) had any such reported associations, which, coupled with extensive supporting evidence in the literature, led to its prioritisation in subsequent analyses.

Evaluation of clinical risk factors in patients on metformin showed that metformin use duration and concomitant PPI use were also associated with vitamin B_12_ deficiency (Table 1). There was a modest increase in model fit for the logistic regression model of metformin-induced vitamin B_12_ deficiency with the rs1801222 SNP included (*R*^2^=0.111) vs without (*R*^2^=0.096).

To determine whether the association with rs1801222 was applicable to individuals of South Asian ancestry, the largest minority ethnic group in the UK, we identified a total of 577 individuals (135 metformin-induced vitamin B_12_-deficient cases and 442 metformin-tolerant controls) (ESM Table 7). In line with primary analyses, individuals with the AA genotype had a higher risk of metformin-induced B_12_ deficiency, with the heterozygotes showing a similar direction of effect (additive model; adjusted *p*=0.0157; OR 1.61 [95% CI 1.09, 2.37] for AG vs GG; OR 2.59 [95% CI 1.20, 5.60] for AA vs GG).

### Secondary analysis of UKB data

To confirm that the rs1801222 association was specific to metformin-induced vitamin B_12_ and not generalised vitamin B_12_ deficiency, we fitted a logistic regression model comparing 577 metformin-induced vitamin B_12_-deficient cases to 3065 non-metformin-induced vitamin B_12_-deficient controls. The inclusion of those individuals excluded from the GWAS owing to lack of metformin dose data, to maximise numbers, accounts for the increase in numbers in the secondary analyses. rs1801222 was again found to be significantly associated with metformin-induced vitamin B_12_ deficiency (additive model; adjusted *p*=0.00511; OR 1.20 [95% CI 1.06, 1.36] for AG vs GG; OR 1.43 [95% CI 1.11, 1.85] for AA vs GG).

A logistic regression model was fitted comparing vitamin B_12_-deficient cases (*n*=3386, of which 555 were metformin-induced) vs non-vitamin-B_12_-deficient controls (373,387, of which 7323 were metformin tolerant), including a binary metformin covariate and an rs1801222 × metformin interaction term (ESM Table 8). A significant interaction was found between rs1801222 genotype and metformin use (additive model; adjusted *p*=0.000249; OR 1.29 [95% CI 1.12,1.47] for interaction with AG genotype; OR 1.65 [95% CI 1.26, 2.16] for interaction with AA genotype).

### Independent replication

We next sought to replicate the association between the rs1801222 A allele and metformin-induced vitamin B_12_ deficiency risk observed in our UKB cohort in three independent clinical cohorts (ESM Table 9). In all cohorts, the rs1801222 genotype distribution conformed to Hardy–Weinberg equilibrium (*p*>0.0001).

#### Scottish cohort

A total of 1,031,090 metformin prescriptions were identified, corresponding to 17,967 distinct individuals with one or more metformin prescription. Among these, 11,567 individuals had genetic data. Based on GP prescribing datasets, 1833 individuals were identified as users of injectable vitamin B_12_ preparations (hydroxocobalamin or cyanocobalamin). Among these, 978 individuals (53.4%) had been treated with metformin at any time prior to receiving the injection.

The rs1801222 genotype distribution in the 44,195 participants from the Scottish cohort was GG 39.7%, AG 46.7% and AA 13.6%. In line with the UKB cohort data (ESM Table 9), vitamin B_12_ deficiency was more prevalent in the AA genotype group treated with metformin vs GG (OR 2.17 [95% CI 1.80, 2.60]; *p*<0.0001). Compared with the GG group, AG individuals also had a higher risk of vitamin B_12_ deficiency in metformin users (OR 1.31 [95% CI 1.13, 1.52]; *p*=0.0004). The effect size was smaller in the non-metformin-exposed GG group (OR 1.28 [95% CI 1.05, 1.56], *p*=0.017). A significant increase in the risk of vitamin B_12_ deficiency for the AA genotype was found in non-metformin treated individuals vs GG genotype (OR 1.28 [95% CI 1.05, 1.56]; *p*=0.017), but not for AG vs GG (OR 1.01 [95% CI 0.87, 1.18]; *p*=0.849) (ESM Table 9).

#### DPP cohort

Low vitamin B_12_ levels (≤203pg/ml) were observed more frequently in the metformin group vs placebo (4.3% vs 2.3%, *p*=0.02) at 5 years (DPPOS year 1) but a statistically significant difference was not observed at 13 years (DPPOS year 9) [3].

In the DPPOS year 1 cohort (5 years of follow-up), rates of vitamin B_12_ deficiency were 1.6%, 3.1% and 6.1% for the GG, GA and AA genotype groups, respectively, in the placebo arm vs 3.1%, 5.2% and 10.2% in the metformin arm (ESM Table 9). The risk of vitamin B_12_ deficiency in the AA genotype group vs GG was significantly higher in the metformin arm (OR 3.58 [95% CI 1.04, 11.06]; *p*=0.022) but not in the placebo arm (OR 3.98 [95% CI 0.62, 16.45]; *p*=0.075). No significant increase was seen for AG vs GG in either the metformin (OR 1.73 [95% CI 0.73, 4.24]) or the placebo arm (OR 1.96 [95% CI 0.61, 6.77]) *p*>0.05).

In DPPOS year 9, vitamin B_12_ deficiency rates were 4.4%, 5.9%, and 4.9% for GG, AG and AA genotype groups in the placebo arm, vs 5.7%, 6.6%, and 18.7% in those respective genotype groups in the metformin arm (ESM Table 9). A significantly increased risk of vitamin B_12_ deficiency was observed in the AA group vs GG in individuals assigned to metformin (OR 3.85 [95% CI 1.62, 9.15]; *p*=0.001) but not for AG vs GG (OR 1.19 [95% CI 0.59, 2.38]; *p*=0.63). There was no significant increase in the risk of vitamin B_12_ deficiency for either the AG or AA genotype groups in individuals assigned to placebo.

#### Liverpool cohort

All 75 individuals in the Liverpool replication cohort were genotyped for the *CUBN* rs1801222 (p.S253F) SNP, with three excluded owing to missing serum vitamin B_12_. Primary outcome was risk of vitamin B_12_ deficiency. Of the 72 included individuals, 28 were classified as vitamin B_12_ deficient (<203 ng/l) and 44 had normal vitamin B_12_ levels (>203 ng/l). There was a statistically significant association between vitamin B_12_ deficiency and rs1801222 AG (OR 3.49 [95% CI 1.20, 10.19]; *p*=0.020) but not AA genotype (OR 1.23 [95% CI 0.26, 5.94]; *p*=0.792) (ESM Table 9).

### Meta-analysis

**Fig. 2:**
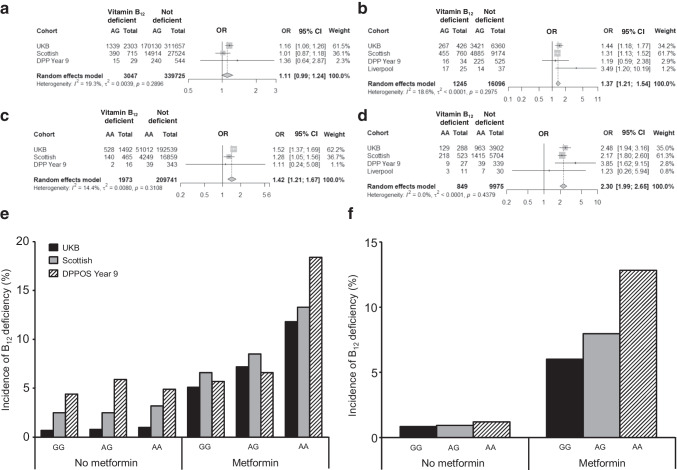
Risk of metformin-induced vitamin B_12_ deficiency is increased by rs1801222 in individuals with the A allele compared with GG. (**a**–**d**) Meta-analysis of the association between vitamin B_12_ deficiency and genotype at the rs1801222 locus in individuals not taking metformin (**a**, **c**) and those on long-term metformin (**b**, **d**). Data are shown for the AG (**a**, **b**) and AA genotypes (**c**, **d**) in the UKB, Scottish and DPPOS year 9 cohorts. We also show data from the Liverpool cohort in the metformin-induced vitamin B_12_ deficiency plots (**b**, **d**). Data represent ORs (95% CI) and *p* values derived from unadjusted χ^2^ analysis. Pooled ORs were determined using a random-effects model. (**e**, **f**) The incidence of vitamin B_12_ deficiency in both users of metformin and those not on metformin, stratified by genotype at the rs1801222 locus, is show for each individual cohort (UKB, Scottish, DPPOS year 9) (**e**) and for the combined cohorts (**f**)

High homogeneity was observed between cohorts for all comparisons (Fig. 2). The observed risk for metformin-induced vitamin B_12_ deficiency demonstrated an additive effect for rs1801222 genotype, with both AG (OR 1.37 [95% CI 1.21, 1.54], *I*^2^=18.6%) and AA (OR 2.30 [95% CI 1.99, 2.65], *I*^2^=0%) genotype groups being at increased risk vs the GG group. An association with vitamin B_12_ deficiency in non-metformin-exposed individuals and genotype was also demonstrated for both AG (OR 1.11 [95% CI 0.99, 1.24], *I*^2^=19.3%) and AA (OR 1.42 [95% CI 1.21, 1.67], *I*^2^=14.4%) genotypes but ORs were lower than in metformin-exposed individuals.

Combined analysis of all the cohorts (Fig. 2) showed that, in non-metformin-exposed individuals, vitamin B_12_ deficiency was seen in 0.84% of the GG genotype group, 0.93% of the AG group and 1.20% of the AA group. Vitamin B_12_ deficiency was more common in metformin-exposed individuals, with an incidence of 6.02% in the GG genotype group, 7.96% in the AG genotype group and 12.84% in the AA genotype group.

### Survival analysis

**Fig. 3:**
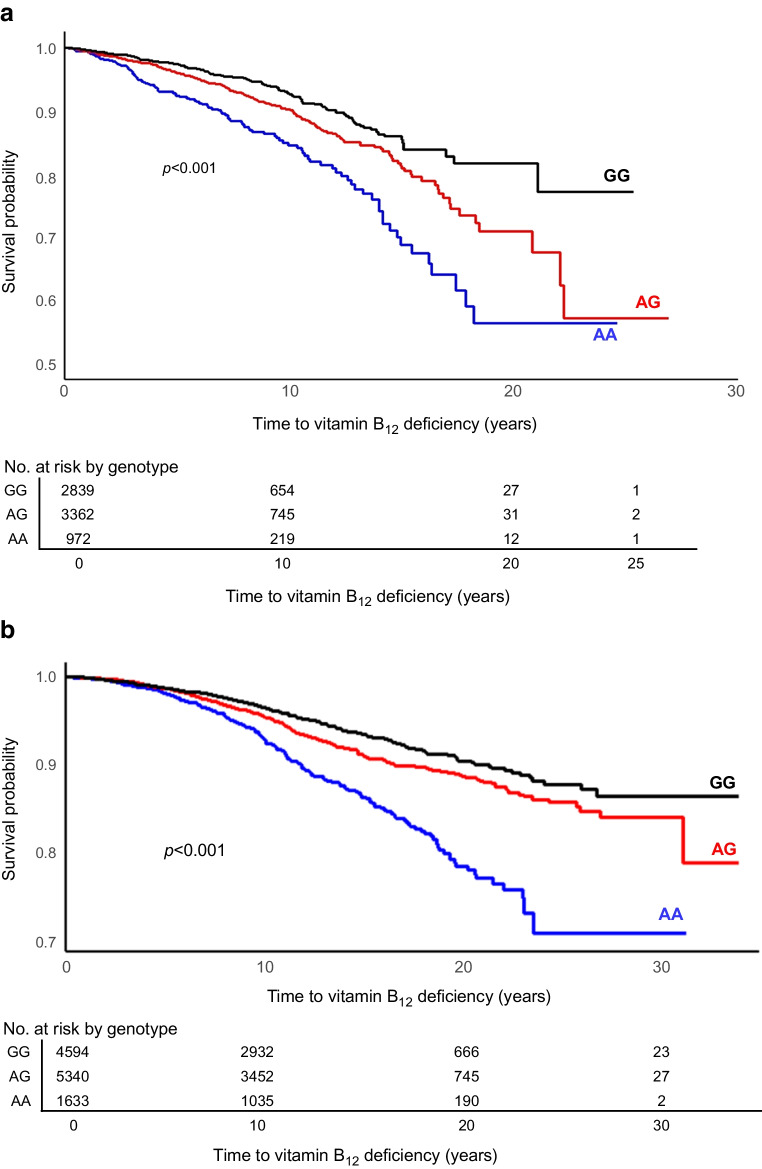
Kaplan–Meier survival analysis of time to first vitamin B_12_ deficiency event in individuals with type 2 diabetes receiving metformin, stratified by the rs1801222 genotype, from the UKB cohort (**a**) and the Scottish replication cohort (**b**)

Using all metformin-exposed individuals in the UKB (metformin-vitaminB_12_-deficient cases *n*=487; metformin-tolerant controls *n*=6686), Kaplan–Meier survival analysis (Fig. 3a) indicated a significant difference in time to metformin-induced vitamin B_12_ deficiency between genotype groups (logrank *p*<0.0001). This observation was replicated in the Scottish cohort (*p*<0.0001) (Fig. 3b).

In an unadjusted Cox regression model, individuals with the AG genotype and those with the AA genotype were at significantly increased risk of vitamin B_12_ deficiency compared with those in the GG genotype reference group (additive model; unadjusted *p*=6.52×10^−11^; HR 1.52 [95% CI 1.34, 1.72] for AG vs GG; HR 2.31 [95% CI 1.80, 2.97] for AA vs GG).

Similarly, in an adjusted Cox regression model, individuals in the AG and AA genotype groups were at significantly increased risk of vitamin B_12_ deficiency compared with individuals in the reference GG genotype group (additive model; adjusted *p*=6.12×10^−11^; HR 1.53 [95% CI 1.35, 1.74] for AG vs GG; HR 2.34 [95% CI 1.81, 3.02] for AA vs GG). This observation was also replicated in the Scottish cohort using an adjusted model for both AG (HR 1.38 [95% CI 1.12, 1.70]; *p*<0.001) and AA (HR 2.15 [95% CI 1.67, 2.76]; *p*<0.001) genotype groups.

### Plasma metformin concentrations

**Fig. 4:**
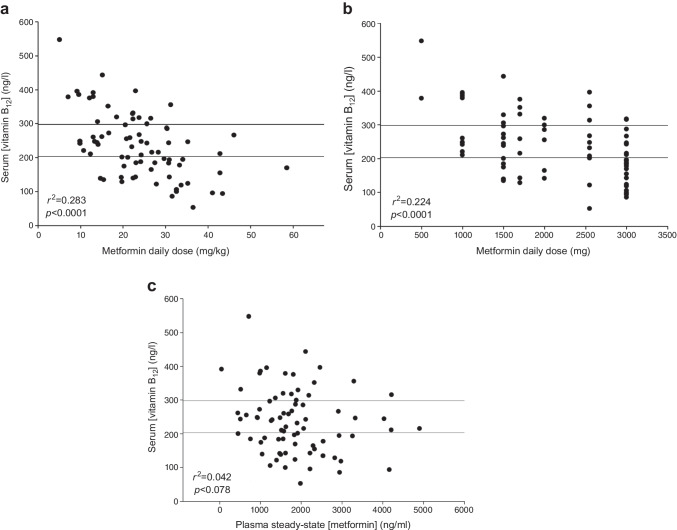
Correlation between serum vitamin B_12_ levels and daily dose of metformin adjusted for mg/kg of body weight (**a**), total daily dose of metformin (**b**) and steady-state metformin plasma levels (**c**) in the Liverpool replication cohort. Normal vitamin B_12_ levels (>298 ng/ml) are defined as values above the top horizontal line; vitamin B_12_ deficiency is defined as values (<203 ng/ml) below the bottom horizontal line; values between the two lines are defined as borderline vitamin B_12_ deficiency (203–298 ng/ml)

Steady-state plasma metformin concentrations, measured in the Liverpool cohort using LC-MS, showed that although serum vitamin B_12_ levels were significantly correlated with both weight-adjusted daily dose (*R*^2^=0.283 *p*<0.0001) and daily dose (*R*^2^=0.224 *p*<0.0001), there was no correlation with steady-state plasma metformin concentrations (Fig. 4).

## Discussion

Using a vitamin B_12_ deficiency phenotype definition, consistent with previous reports [1, 2], there were 487 (6.8%) deficient individuals of White European ancestry receiving metformin in the UKB cohort out of a total of 7173. Our data show a significant association between metformin-induced vitamin B_12_ deficiency risk and a non-synonymous SNP (rs1801222) in the *CBN* gene, which encodes the cubilin receptor (ESM Table 6, Fig. 1). This association was replicated in two independent cohorts of individuals with type 2 diabetes (Fig. 3 and ESM Table 9). Although statistical significance was not observed in the Liverpool replication cohort because of its small sample size, the direction of effect was consistent with the primary analysis. The A allele of the rs1801222 variant has previously been reported to be associated with vitamin B_12_ levels [12–15]. However, the novelty of our data is that they show an interactive effect of metformin exposure with the risk of vitamin B_12_ deficiency in individuals with the A allele (Fig. 3). This conclusion is strengthened first by the secondary analyses, which show the effect of metformin on vitamin B_12_ deficiency, and second by replication in independent cohorts.

Clearly, it is well known that many individuals with vitamin B_12_ deficiency are clinically asymptomatic and therefore the overall rate of vitamin B_12_ deficiency identified in our analyses may be an underestimate. A limitation of our study is that we have focused on vitamin B_12_ deficiency without measuring other biomarkers that are associated with vitamin B_12_ status, such as homocysteine, methylmalonic acid and holo-transcobalamin. Although this was not possible in UKB, this is an area for further study in cohorts where samples are available.

Previous work highlighted the role of the cubilin receptor in intestinal vitamin B_12_ absorption [27]. Free duodenal vitamin B_12_ binds to intrinsic factor (IF) [28], with the vitamin B_12_–IF complex binding in a calcium-dependent manner to the cubilin receptor on enterocytes in the distal ileum, resulting in receptor-mediated endocytosis and vitamin B_12_ absorption. Metformin has been postulated to antagonise calcium cations and interfere with calcium-dependent IF–B_12_ complex binding to cubilin [29] and therefore with vitamin B_12_ absorption. We propose that this interference by metformin may be enhanced in individuals with the A allele in the cubilin gene, though mechanistic investigations are needed to prove this. However, our hypothesis is also consistent with the fact that for the first time we show that while vitamin B_12_ deficiency was closely associated with metformin dose, consistent with previous studies [1, 8], it was not associated with plasma concentrations of metformin (Fig. 4), supporting the concept that gut absorption (and its dysfunction) plays a crucial role in metformin-induced vitamin B_12_ deficiency. This is also consistent with the fact that metformin’s primary beneficial effect in type 2 diabetes is in the intestines [30].

We also show the same association in South Asian individuals with type 2 diabetes on metformin in the UKB. The population frequency of the rs1801222A allele is 20%, ranging from 15% in East Asians to 29% in Europeans [31], suggesting that the variant will impact all population groups treated with metformin.

We focused on individuals with type 2 diabetes on metformin. However, metformin is also used for prevention of type 2 diabetes, in polycystic ovary syndrome and long COVID; it has been trialled in cancer prevention and as an anti-senescent drug [32]. Our data, consistent with the literature [32], show that chronic duration of therapy, higher cumulative metformin dose and concomitant PPI therapy (Table 1) increase the risk of vitamin B_12_ deficiency. We can now add the rs1801222 A allele as an additional risk factor. This is likely to be important in diseases beyond type 2 diabetes where metformin is used chronically. The genetic association observed appears to be equally applicable regardless of gender, though there was a significantly higher percentage of female participants in the case vs control group (Table 1). Therefore, no analysis of the data was undertaken stratifying by gender as the genetic association. Clearly this is a limitation but would require a larger, adequately statistically powered study to examine.

What are the clinical implications of our findings? Although many authors, and regulators, have highlighted the importance of checking vitamin B_12_ levels in patients on metformin and giving replacement therapy (which is easy, cheap and readily available) when appropriate, monitoring is rarely done. Furthermore, most monitoring guidelines, including both the MHRA in the UK [9] and the ADA [33], suggest the need for ‘periodic’ vitamin B_12_ monitoring but do not provide a monitoring frequency, which probably leads to lack of monitoring. Our data clearly shows that vitamin B_12_ deficiency occurs earlier (within a few years) in individuals with the AA genotype at the rs1801222 locus in the cubilin receptor gene (Fig. 3). The magnitude of this genetic interaction with metformin exposure is large and clinically relevant: 14% of the population with the AA genotype are ~2.5 times more likely than the wild-type GG group to require vitamin B_12_ replacement therapy in the UKB and Scottish cohorts. Where vitamin B_12_ deficiency is based upon measured vitamin B_12_ levels rather than treatment, as in the DPPOS, the odds increase to ~3.8-fold at 9 years. When followed up from initiation of metformin, 10% of individuals with the AA genotype will be vitamin B_12_ deficient by 11 years compared with 21 years for the GG genotype group. Thus, where genotype is known, there is a strong case for initiating serum vitamin B_12_ monitoring from initiation of metformin treatment in people with the AA genotype but deferring this until 10 years of treatment in the rest of the population who have no other risk factors. However, as genotyping is not currently routinely available and serum vitamin B_12_ measurement is cheap, our results reinforce the causal association of metformin treatment with vitamin B_12_ deficiency and guideline recommendations for routine screening for vitamin B_12_ deficiency in people treated with metformin.

In conclusion, we describe a novel additive effect on vitamin B_12_ deficiency risk for metformin exposure with a previously reported genetic risk factor in the cubilin receptor gene. Indeed, individuals with the A allele are at significantly higher risk earlier during metformin therapy. Although in vitro and human functional studies are required to elucidate how the combination of metformin and this genetic variant impact vitamin B_12_ absorption, the genetic variant may have clinical utility in stratifying monitoring of vitamin B_12_ levels in patients on chronic metformin treatment.

## Supplementary Information

Below is the link to the electronic supplementary material.

ESM (PDF 879 KB)

ESM Table 2 (XLSX 59.5 KB)

## Data Availability

All data are available upon request from the authors and summary data will be made available via the UK Biobank (https://www.ukbiobank.ac.uk/projects/).

## Abbreviations

DPPOS: Diabetes Prevention Program Outcomes Study
GWAS: Genome-wide association study
IF: Intrinsic factor
MHRA: Medicines and Healthcare products Regulatory Agency
PPI: Proton pump inhibitor
QC: Quality control
UKB: UK Biobank
