# Supplementary material for: Identification of a genetic risk factor for metformin-induced vitamin B12 deficiency
Source: Diabetologia. 2026 Jan 15;69(4):953–65. doi: 10.1007/s00125-025-06655-5 (PMC12957035; doi:10.1007/s00125-025-06655-5)
Supplement: Supplementary file 1 — ESM (PDF 879 KB) [file 125_2025_6655_MOESM1_ESM.pdf]

ELECTRONIC SUPPLEMENTARY MATERIALS

IDENTIFICATION OF A GENETIC RISK FACTOR FOR METFORMIN-INDUCED VITAMIN B12 DEFICIENCY

#Faye D. Baldwin[1], # Khaled F. Bedair[3], Andrea L. Jorgensen[1], Lewis Green[2], Innocent G. Asiimwe[1], Colin N.A. Palmer[3], Archie Campbell[9], Caroline Hayward[9.10], Qing Pan[4], Shiyu Shu[4], Josephine H. Li[5,6,7,8], Diabetes Prevention Program (DPP) Research Group, *Ewan R. Pearson[3], *Munir Pirmohamed[2], *Daniel F. Carr[2].

METHODS

Covariate Selection

Covariates including sex, month and year of birth were obtained from UKB data-fields 31, 52 and 34, and date of birth was approximated using data-fields 52 and 34. Age at first metformin prescription was calculated by the difference in date of birth and first recorded metformin prescription, using GP prescription records (UKB data-field 42039). Duration of metformin use was calculated by the difference in first and last recorded metformin prescription dates. Use of PPIs 6 months was calculated by identifying PPI prescriptions 6 months (182.5 days) prior to index date in cases (6 months prior first recorded vitamin B12 diagnosis date or B12 injection prescription) and controls (6.88 years post first recorded metformin prescription). Cumulative dose of metformin (mg) was calculated by multiplying drug strength by the corresponding quantity of each prescription, then summing across all prescriptions, with reference to the corresponding strength of unique drug names, using GP prescription records. To adjust for population substructure, the first two principal components (PCs) were obtained from UKB data-field 22009. Variables were chosen based on priori knowledge of putative association with vitamin B12 deficiency and were adjusted for in primary analysis logistic regression.

UKB Genotyping

Imputed genotypes from chromosomes 1-22 (version 3 .bgen) were downloaded. In addition to UKB-applied QC, we excluded variants with a minor allele frequency (MAF) < 0.01, missing genotype rate > 0.05, Hardy-Weinburg equilibrium (HWE) test p-value < 10^{-5} and imputation quality (INFO score) < 0.3 prior to association analysis, using PLINK2 [1].

Per-participant quality control

Prior to conducting genetic association analysis, the following participant-level exclusion criteria were applied: removal of individuals with self-reported non-White British ancestry, discrepancies between self-reported and genetic sex, presence of sex chromosome aneuploidy, outliers in heterozygosity and missingness rates, and individuals inferred to be related (third-degree or closer) based on a kinship threshold of 0.0884.

Scottish Replication Cohort

Generation Scotland (GS): The study's unique features include family-based recruitment to capture family groups, extensive phenotype data such as cognitive function, personality traits, and mental health, and broad consent for linkage to routine healthcare records. Participants also consented to the reuse of their data and samples in future medical research and for potential re-contact. These features enhance the study's capacity to investigate genetic and environmental factors associated with a wide range of illnesses and risk factors, ensuring its long-term value for research.

GoDARTS: Baseline data collection involved lifestyle questionnaires covering physical activity, smoking history, and menopausal history for women, alongside clinical observations and biological samples. These data are linked to NHS records, enabling access to morbidity, mortality, and prescribing information for long-term follow-up. Additionally, 95% of participants provided consent for re-contact, enabling their involvement in future studies.

SHARE: Consent was obtained from individuals to use their NHS data to assess their suitability for research projects and allows for re-contact to participate in studies. Additionally, participants can consent to have leftover blood samples from routine venepuncture stored for future research purposes, further expanding the utility of the resource.

Liverpool Replication Cohort

DNA extraction and Genotyping: Genomic DNA was extracted from 5ml whole blood using Chemagic Magnetic Separation Module I platform (Chemagen Auto-Q Biosciences Germany) according to the manufacturer's instructions. DNA (20 ng) was genotyped for the SNPs identified as statistically significant in genome-wide analysis using a validated TaqMan SNP genotyping assay (C___2822674_10) (Applied Biosystems Paisley UK) on a QuantStudio 6 real-time PCR platform (Applied Biosystems).

Serum biochemical analysis: Analysis of serum vitamin B12 was performed on an Access 2 immunoassay analyser using manufacturer's reagents and protocols (Beckman Coulter Inc. Fullerton CA). Based on previous literature [2,3] vitamin B12 deficiency was defined as serum concentration ≤203 ng/mL (150 pmol/L).

Measurement of plasma metformin

Preparation of QC samples and standards: All concentrations of metformin and phenformin refer to their hydrochloric salts. Stock solutions of metformin (1 mg/mL) and internal standard (IS) phenformin (1 mg/mL) were prepared by dissolving the hydrochloride salts in mobile phase A which comprised acetonitrile 5 mM ammonium acetate formic acid (90:10:0.1 v/v/v; pH 5.1). A series of metformin standard solutions at different concentrations (0.3-10000 ng/mL) were prepared through serial dilutions of the stock concentration with mobile phase A. High medium and low and LLOQ QC concentrations of 300, 30, 3 and 0.3 ng/mL respectively were chosen and prepared. Metformin concentrations are quoted as their salt (metformin·HCl) free base concentrations were calculated by dividing the salt concentration by 1.28. All working solutions were stored at +4°C and used within 24 hours of preparation.

Extraction: To 50 µL of plasma 2.5 µL of the IS (10 µg/mL) and metformin (appropriate concentrations) working solutions were added and vortexed for 5 sec. Mobile phase A (447.5 µL) was added vortexed and left for 15 min at room temperature to ensure complete protein precipitation had occurred. The solution was then centrifuged at 14000 rpm (17500×*g*) for 10 min at room temperature. The supernatant was transferred to a clean container and centrifuged again under the same conditions to minimise precipitate being transferred prior to analysis by LC-MS/MS.

Chromatography: Chromatography was performed using a Phenomenex Luna HILIC column (250 mm x 4.6 mm i.d. 5µM) connected to a HILIC guard cartridge (4 mm x 3 mm). A gradient mobile phase was used consisting of gradient mobile phase A and mobile phase B (5 mM ammonium acetate formic acid (100:0.1 v/v) pH 5.1). Mobile phase A was held at 80% for 2 min then 50% for 5 min then returned at

80% for 5 min equilibration. A 10 µL sample was used for detection. The total run time was 12.0 min. The flow rate was maintained at 0.6 mL/min. The autosampler temperature was set to 4°C and the column remained at room temperature (23°C). To establish possible matrix ionisation effects, we used post plasma precipitation extracts from blank plasma samples and monitored any differences in electron spray ionisation responses for both metformin and the IS through a post-column infusion. The results revealed that both retention times and response appeared satisfactory.

MS/MS detection*:* Detection was performed on an API 3000 LC-MS/MS (Applied Biosystems). The operating conditions were: ionisation positive mode; source temperature 450°C; ion spray 5000v; Nitrogen gas (99.999%) at 3 10 and 14 psi as the collision gas curtain gas and nebuliser gas respectively. Optimised parameters for metformin and phenformin were: entrance potential (EP) 10v; declustering potential (DP) 31 and 36v; focusing potential (FP) 130v; collision energy (CE) 27 and 29v; collision cell exit potential (CXP) 12 and 10v respectively. Analytes were detected using multiple reaction monitoring (MRM) with transitions of *m/z* for metformin and phenformin monitored at 130.14→70.9 and 206.15→59.9 respectively (Appendix 2.7.1) with a dwell time of 200ms per transition. Data acquisition was performed with Analyst™ (v.4.2) on a MS workstation.

RESULTS

ESM Table 1 Clinical codes used for phenotype classification in UKBB

Type 2 Diabetes

Data source/identification or code	Clinical code type	Clinical code	Description of code
UKBB Data-Field 130708: Date E11 first reported (non-insulin-dependent diabetes mellitus) UKBB Data-Field 130709: Source of report of E11 (non-insulin-dependent diabetes mellitus)	Read_2<br>Read_3<br>ICD10<br>ICD9	Mapping first occurence of clinical codes (read_2 read_3 ICD9 ICD10) for diagnosis of T2D	Includes date when diagnosed and source of diagnosis - as we only want accurate diagnoses of T2D we omit self-reported and death-register data as these diagnosis dates may be delayed. We used Data-Field 130709 (Source of E11 report) to identify only individuals with T2D code recorded in either primary care only or hospital admissions data only. If an individual has diagnosis date recorded in both hospital admissions and primary care we take the earliest diagnosis date as the individual's diagnosis date.

Vitamin B12 Deficiency

Data source/identification or code	Clinical code type	Clinical code
UKBB 42040 GP clinical records and use of UKB_participants_with_a_given_condition.	ICD10	D511 (Vitamin B12 deficiency anemia due to selective vitamin B12 malabsorption with proteinuria)<br>D513 (Other dietary vitamin B12 deficiency anemia)<br>D518 (Other specified vitamin B12 deficiency anemias)<br>D519 (Vitamin B12 deficiency unspecified)
	ICD9	2811 (Other vitamin B12 deficiency anaemia)

	Read 2	D0111 (Vitamin B12 deficiency anemia due to selective vitamin B12 malabsorption with proteinuria)<br>D0110 (Vitamin B12 deficiency anaemia due to dietary causes) D011. (Other vitamin b12 deficiency anemias)<br>D011X (Vitamin B12 deficiency anaemia unspecified)<br>D011Z (Other vitamin B12 deficiency anaemia NOS)<br>C2621 (B12 DEFICIENCY NOS)
	Read 3	C2621 (Vit B12 Deficiency)<br>D0111 (VitB12 def anemia due to selective vitamin B12 malabsorption with proteinuria)<br>Dyu06 (Vitamin B12 deficiency anaemia unspecified)<br>D011Z (Other vitamin B12 deficiency anaemia NOS<br>XE13g (Other vitamin B12 deficiency anaemias)<br>Xa9Aw (Vitamin B12 deficiency anaemia)<br>XE13h (Vitamin B12 deficiency anaemia due to dietary causes)

Pernicious Anemia

**Data source/identification or code**	**Clinical code type**	**Clinical code**
UKBB 42040 GP clinical records and use of UKB_participants_with_a_given_condition.	ICD10	D510 (Pernicious anemia)
	ICD9	2810 (Pernicious anemia)
	Read 2	D010. (includes pernicious anemia Addison's anaemia Biermer's congenital pernicious anaemia and congenital deficiency of intrinsic factor)
	Read 3	XE2ro

ESM Table 2. Prescribing Codes used to identify patients in the UKBB.

ESM Table 2 is provided as a sperate Excel (.xlsx) file

ESM Table 3. Approved names were used to identify and extract metformin-treated patients (17,967) from the GP prescription records in the Scottish replication cohort.

Approved Name	Formatted BNF code	Prescriptions	%
**ALOGLIPTIN AND METFORMIN HYDROCHLORIDE**	6.1.2.3	2448	0.24%
**LINAGLIPTIN AND METFORMIN HYDROCHLORIDE**	6.1.2.3	23	0.00%
**METFORMIN HYDROCHLORIDE**	6.1.2.3	1014021	98.34%
**PIOGLITAZONE AND METFORMIN HYDROCHLORIDE**	6.1.2.3	5586	0.54%
**ROSIGLITAZONE WITH METFORMIN HYDROCHLORIDE**	6.1.2.3	6935	0.67%
**SAXAGLIPTIN AND METFORMIN HYROCHLORIDE**	6.1.2.3		0.00%
**SITAGLIPTIN AND METFORMIN HYDROCHLORIDE**	6.1.2.3	1293	0.13%
**VILDAGLIPTIN AND METFORMIN HYDROCHLORIDE**	6.1.2.3	784	0.08%
**Total**		1031090	100.00%

ESM Table 4. Approved names used to identify and extract B12 injection of the metformin treated patient (1,365, 978) from the GP prescription records in the Scottish replication cohort

Approved Name	Formatted BNF code	Prescriptions	%
**CYANOCOBALAMIN**	9.1.2	555	5.8
**HYDROXOCOBALAMIN**	9.1.2	9160	94.2
**Total**		9,725	100

ESM Table 5. Liverpool replication cohort (n=75) summary clinical data.

	Mean (SD)	Range	R_2	P
Demography<br>Age (years)	64 (9.7)	42.3 - 80.6		
BMI (kg/m^2)	32.6 (5.1)	22.2 - 43.6	0.058	0.038
Biochemistry & Haematology<br>Vitamin B_{12} (ng/mL)	237 (96)	52 – 547	-	-
Folate (µg/ml)	8.6 (4.8)	1.6 – 20	0.041	0.082
Lactate (mmol/L)	2.10 (0.75)	0.8 – 3.9	-0.043	0.077
Haemoglobin (g/L)	1.32 (0.16)	0.88 – 1.61	0.006	0.494
Haematocrit (%)	38.9 (4.6)	26 – 48.2	0.003	0.626
Mean Corpuscle volume (Fl)	88.4 (4.8)	74.9 – 99.3	0.018	0.254
Kidney function<br>Creatinine (µmol/L)	90.4 (27.8)	75 - 195	0.041	0.08
Urea (mmol/L)	6.8 (2.2)	3.3 - 13.6	0.007	0.49
CL_{CR} (mL/min)	90.8 (33)	37 - 174	<0.001	0.913
eGFR (mL/min)	74 (21)	33 - 138	-0.012	0.355
Liver function<br>Albumin (g/L)	42 (3.5)	32 – 48	<0.0001	0.961
Alanine aminotransferase (g/L)	25 (15)	10 - 117	<0.0001	0.905
Alkaline phosphatase (g/L)	76 (24)	40 – 167	<0.0001	0.968
Gamma GT (U/L)	36 (31)	7 - 171	0.023	0.196
Bilirubin (µmol/L)	9 (5)	3 - 34	0.009	0.413
Metformin parameters<br>Metformin total daily dose (mg)	2125 (793)	500 – 3000	-0.224	<0.001
Metformin single dose (mg)	793 (222)	500 – 1000	-0.135	0.001
Metformin total daily dose (mg/kg)	24 (10)	5 - 59	-0.283	<0.001
Metformin cumulative dose (kg)	6.99 (6.31)	0.15 – 31.24	-0.127	0.002
Metformin plasma level (ng/mL)	1894 (1027)	30 - 5387	-0.042	0.078

**Duration on metformin (years)**	8.05 (6.04)	0.41 – 29.03	-0.079	0.015
**Duration of T2DM (years)**	10.17 (6.85)	0.41 – 31.67	-0.003	0.644

P-value and R2 of univariate analysis of vitamin B12 levels are shown.

ESM Table 6. Statistically significant SNPs from the discovery cohort of the metformin-induced vitamin B12 deficiency genome-wide association study

							Cases (n=487)				Controls (n=6686)				MAF				
**SNP**	**Chr**	**BP (GRCh37)**	**Gene loci**	**Functional Effect**	**A1**	**A2**	**A1/A1**	**A1/A2**	**A2/A2**	**Miss**	**A1/A1**	**A1/A2**	**A2/A2**	**Miss**	**All**	**Cases**	**Con**	**Additive OR (95% CI)**	**P**
**rs6661844**	1	64870696	Intergenic	-	T	G	44	211	231	1	375	2372	3923	16	0.24	0.31	0.23	0.70 (0.61,0.81)	$3.84x10^{-6}$
**rs12740521**	1	168609604	*LOC105371604*	Intronic	G	A	14	117	356	0	276	2270	4140	0	0.21	0.15	0.21	1.57 (1.30,1.89)	$8.05x10^{-7}$
**rs35832336**	2	233411708	*CHRNG*	3′ UTR	G	C	44	207	229	7	367	2421	3794	104	0.24	0.31	0.24	0.69 (0.60,0.80)	$1.79x10^{-6}$
**rs111956957**	6	40324392	*LRFN2*	3' Downstream	A	C	0	0	482	5	0	176	6460	50	0.01	0.00	0.01	$9.47x10^{4}$ (1.62-Inf)	$2.16x10^{-6}$
**rs1801222**	10	17156151	*CUBN*	Non-synonymous	G	A	143	232	112	0	2696	3130	860	0	0.37	0.47	0.36	1.56 (1.36,1.79)	*$1.86x10^{-10}$
**rs4750496**	10	14284606	*FRMD4A*	Intronic	G	A	302	158	24	3	3388	2696	577	25	0.28	0.21	0.29	0.67 (0.57,0.79)	$9.20x10^{-7}$
**rs2245007**	14	54007026	*LOC105370504*	Intronic	G	A	183	221	76	7	1920	3233	1406	127	0.46	0.39	0.46	0.72 (0.63,0.83)	$4.84x10^{-6}$
**rs6496476**	15	88900454	*NTRK3-AS1*	Intronic	T	C	100	240	144	3	1902	3324	1403	57	0.47	0.55	0.46	1.42 (1.24,1.63)	$4.43x10^{-7}$
**rs3829509**	16	87903082	*SLC7A5*	5′ UTR	C	G	10	121	326	30	267	2130	4016	273	0.20	0.15	0.21	1.56 (1.29,1.89)	$1.96x10^{-6}$
**rs485073**	19	49207255	*FUT2*	3′ UTR	G	A	100	266	117	4	1970	3338	1334	44	0.46	0.51	0.45	1.39 (1.21,1.59)	$2.99x10^{-6}$

Where applicable, SNPs represent the most statistically significant SNP at that locus. *P-values passing the genome-wide significance threshold ($p<5x10^{-8}$).

ESM Table 7. UK biobank cohort demographics and clinical characteristics included in the logistic regression analysis for rs1801222 limited to individuals of self-reported South-Asian ancestry.

Variable	Case (n=135)	Control (n=442)	OR (95% CI)	p
Gender (Female) n (%)	64 (47%)	168 (38%)	0.75 (0.4,-1.15)	0.051
Age (yrs) at first metformin prescription	55 ± 7	56 ± 9	-	0.064
Time to B12 event (yrs)	6.9 ± 4.4	-	-	-
Cumulative Metformin Dose (mg)	$5.88 \times 10^7 \pm 3.9 \times 10^7$	3.78x107 ± 3.4x107	-	<0.001
Duration of Metformin Use (yrs)	10.2	6.7	-	<0.001
Proton Pump Inhibitor use (6 months prior)	68 (50%)	77 (17%)	4.20 (2.7,-6.54)	<0.001

Data represent mean and SD. Statistical significance determined by Pearson's Chi-squared test of Welch's two sample T-test.

ESM Table 8. Logistic regression analysis of vitamin B12 deficient vs non B12 deficient individuals in the full UK biobank cohort including a binary variable for metformin use and a SNP x metformin use interaction variable**.**

**Variable**		**OR (95% CI)**	**p**
Gender	Female	-	-
	Male	0.91 (0.85,0.97)	0.004880*
Metformin Use		7.85(6.71,9.16)	< 2e-16*
rs1801222		1.29(1.12,1.47)	1.40e-13*
Principal component 1		1.00 (0.98,1.03)	0.688002*
Principal component 2		1.00 (0.98,1.03)	0.533696
Principal component 3		0.99 (0.97,1.01)	0.304971
Principal component 4		1.02 (1.00,1.03)	0.064222
Principal component 5		1.03 (1.02,1.04)	2.09e-15
Principal component 6		0.99 (0.97,1.01)	0.244481
Principal component 7		1.00 (0.98,1.02)	0.796038
Principal component 8		1.00 (0.98,1.02)	0.817186
Principal component 9		1.00 (0.99,1.01)	0.345409

Principal component 10	1.00 (0.99,1.02)	0.449081
rs1801222 x metformin	1.29 (1.12,1.47)	0.000249

* Represents statistically significant associations

ESM Table 9. Incidence of metformin and non-metformin-induced B12 deficiency by rs1801222 genotype across the UK Biobank and replication cohorts.

			rs1801222			AG vs GG		AA vs GG	
**Metformin**	**B12 Deficiency**	**N**	**GG**	**AG**	**AA**	**OR (95% CI)**	**p**	**OR (95% CI)**	**p**
**UK Biobank Cohort**									
**No**	No	362678 (99.2%)	141527 (99.3%)	170130 (99.2%)	51,012 (99.0%)				
	Yes	2831 (0.8%)	964 (0.7%)	1,339 (0.8%)	528 (1.0%)	1.16 (1.06,1.26)	0.0006*	1.52 (1.37,1.69)	<0.0001*
**Yes**	No	7323 (92.3%)	2939 (94.9%)	3421 (92.8%)	963 (88.2%)				
	Yes	555 (7.0%)	159 (5.1%)	267 (7.2%)	129 (11.8%)	1.44 (1.18,1.77)	0.0004*	2.48 (1.94,3.16)	<0.0001*
**Scottish Cohort**									
**No**	No	31773 (97.4%)	12610 (97.5%)	14914 (97.5%)	4249 (96.8%)				
	Yes	855 (2.6%)	325 (2.5%)	390 (2.5%)	140 (3.2%)	1.01 (0.87,1.18)	0.849	1.28 (1.05,1.56)	0.017*
**Yes**	No	10589 (91.5%)	4289 (93.4%)	4885 (91.5)	1415 (86.7%)				
	Yes	978 (8.5%)	305 (6.6%)	455 (8.5%)	218 (13.3%)	1.31 (1.13,1.52)	0.0004*	2.17 (1.80,2.60)	<0.0001*
**DPPOS Year 1 Cohort (5 years after randomisation)**									
**No**	No	692 (97.5%)	366 (98.4%)	280 (96.9%)	46 (93.9%)				
	Yes	18 (2.5%)	6 (1.6%)	9 (3.1%)	3 (6.1%)	1.96 (0.61,6.77)	0.199	3.98 (0.62,16.45)	0.075

**Yes**	No	675 (95.5%)	348 (6.9%)	274 (94.8%)	53 (89.8%)				
	Yes	32 (4.5%)	11 (3.1%)	15 (5.2%)	6 (10.2%)	1.73 (0.73,4.24)	0.17	3.58 (1.04,11.06)	0.022*
**DPPOS Year 9 Cohort (13 years after randomisation)**									
**No**	No	583 (95.0%)	304 (95.6%)	240 (94.1%)	39 (95.1%)				
	Yes	31 (5.0%)	14 (4.4%)	15 (5.9%)	2 (4.9%)	1.36 (0.64,2.87)	0.422	1.11 (0.24,5.08)	0.889
**Yes**	No	564 (92.9%)	300 (94.3%)	225 (93.4%)	39 (81.3%)				
	Yes	43 (7.1%)	18 (5.7%)	16 (6.6%)	9 (18.7%)	1.19 (0.59,2.38)	0.632	3.85 (1.62,9.15)	0.001*
**Liverpool cohort***									
**No**	-	-	-	-	-	-	-	-	**-**
	-	-	-	-	-	-	-	-	**-**
**Yes**	No	44	23 (74.2%)	14 (45.2%)	7 (70.0%)				
	Yes	28	8 (25.8%)	17 (54.8%)	3 (30.0%)	3.49 (1.20,10.19)	0.020*	1.23 (0.26,5.94)	0.792

Data for DPPOS is shown at both 5 years and 13 years follow-up. Odds ratios are calculated for B12 treatment for A/G and A/A genotype groups by unadjusted Chi-squared test. # Represents statistically significant results ($p<0.05$). *Liverpool cohort had only metformin exposed individuals and B12 deficiency is defined by measured serum B12 levels.

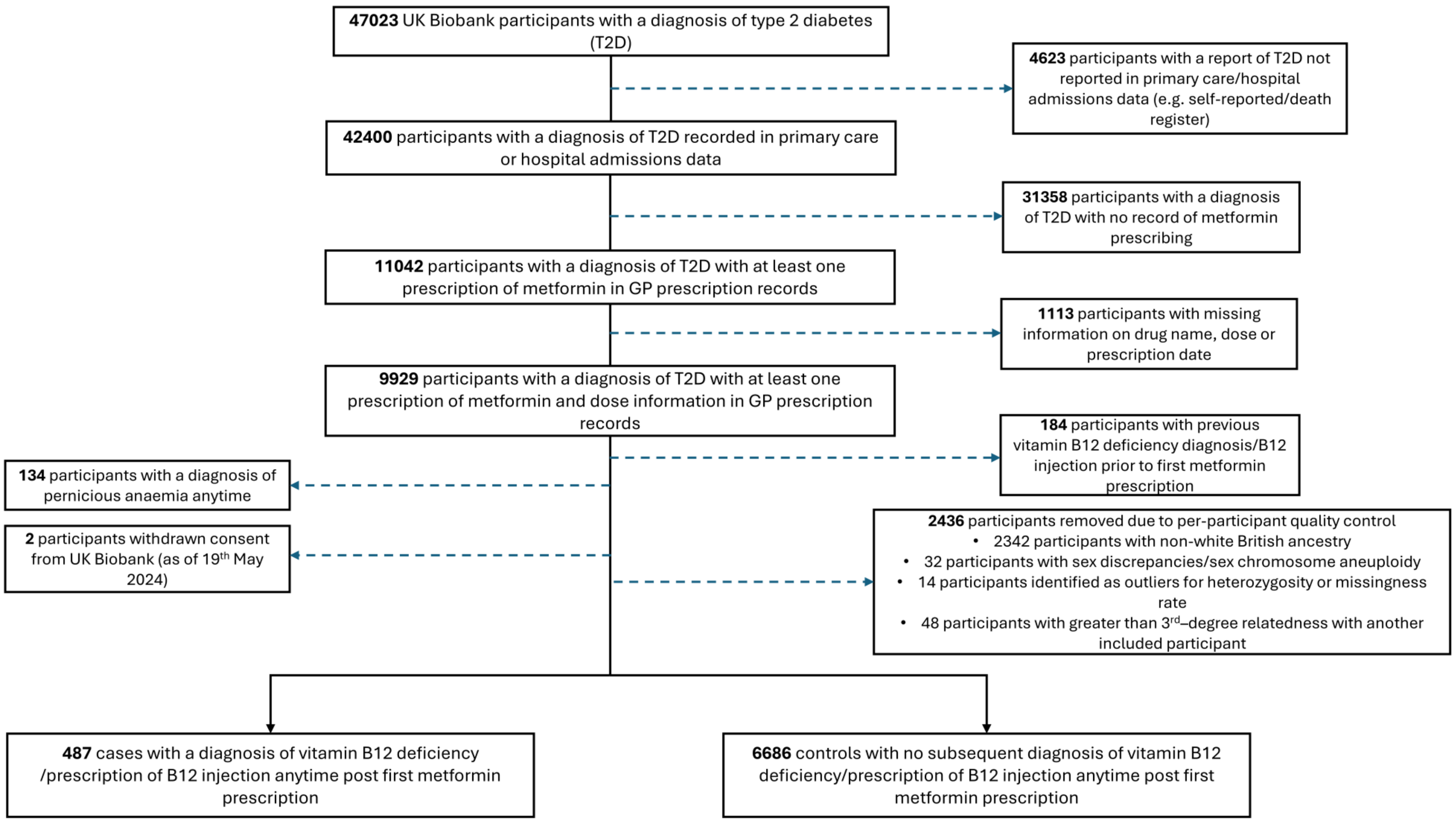

ESM Figure 1. Flow diagram showing how the metformin B12 deficiency cases and controls analysed in the genome-wide association study were identified.

References

1. Chang CC, Chow CC, Tellier LC, Vattikuti S, Purcell SM, Lee JJ. Second-generation PLINK: rising to the challenge of larger and richer datasets. Gigascience 2015; 4: 7.

2. de Jager J, Kooy A, Lehert P, et al. Long term treatment with metformin in patients with type 2 diabetes and risk of vitamin B-12 deficiency: randomised placebo controlled trial. Bmj 2010; 340: c2181.

3. Lindenbaum J, Savage DG, Stabler SP, Allen RH. Diagnosis of cobalamin deficiency: II. Relative sensitivities of serum cobalamin, methylmalonic acid, and total homocysteine concentrations. American journal of hematology 1990; 34(2): 99-107.

DPP, DPPOS 1, DPPOS 2 & DPPOS 3A Research Group (1996-2021)

* denotes Principal Investigator
** denotes Program Coordinator

Pennington Biomedical Research Center (Baton Rouge, LA)

George A. Bray, MD*
Kishore M. Gadde, MD*
Iris W. Culbert, BSN, RN, CCRC**
Jennifer Arceneaux RN, BSN**
Annie Chatellier, RN, CCRC**
Amber Dragg RD, LDN**
Catherine M. Champagne, PhD, RD
Crystal Duncan, LPN
Barbara Eberhardt, RD, LDN
Frank Greenway, MD
Fonda G. Guillory, LPN
April A. Herbert, RD
Michael L. Jeffirs, LPN
Betty M. Kennedy, MPA
Erma Levy, RD
Monica Lockett, LPN
Jennifer C. Lovejoy, PhD
Laura H. Morris, BS
Lee E. Melancon, BA, BS
Donna H. Ryan, MD
Deborah A. Sanford, LPN
Kenneth G. Smith, BS, MT
Lisa L. Smith, BS
Julia A. St.Amant, RTR
Richard T. Tulley, PhD
Paula C. Vicknair, MS, RD
Donald Williamson, PhD
Jeffery J. Zachwieja, PhD

University of Chicago (Chicago, IL)

Kenneth S. Polonsky, MD*
Janet Tobian, MD, PhD*
David A. Ehrmann, MD*
Margaret J. Matulik, RN, BSN**
Karla A. Temple, PhD, RDN, LDN**
Bart Clark, MD
Kirsten Czech, MS
Catherine DeSandre, BA
Brittnie Dotson, MS
Ruthanne Hilbrich, RD
Wylie McNabb, EdD
Ann R. Semenske, MS, RD
Celeste C. Thomas, MD

Jefferson Medical College (Philadelphia, PA)

Jose F. Caro, MD*
Kevin Furlong, DO*
Barry J. Goldstein, MD, PhD*
Pamela G. Watson, RN, ScD*
Kellie A. Smith, RN, MSN**
Jewel Mendoza, RN, BSN**
Marsha Simmons, CCRP**
Wendi Wildman, RN**
Renee Liberoni, MPH
John Spandorfer, MD
Constance Pepe, MS, RD

University of Miami (Miami, FL)

Richard P. Donahue, PhD*
Ronald B. Goldberg, MD*
Ronald Prineas, MD, PhD*
Jeanette Calles, MSEd**
Anna Giannella, RD, MS**
Patricia Rowe, MPA**
Juliet Sanguily, RN**
Paul Cassanova-Romero, MD
Sumaya Castillo-Florez, MPH
Hermes J. Florez, MD
Rajesh Garg, MD
Lascelles Kirby, MS
Olga Lara
Carmen Larreal
Valerie McLymont, RN
Jadell Mendez
Arlette Perry, PhD
Patrice Saab, PhD
Bertha Veciana

The University of Texas Health Science Center (San Antonio, TX)

Steven M. Haffner, MD, MPH*
Helen P. Hazuda, PhD*
Maria G. Montez, RN, MSHP, CDE**
Kathy Hattaway, RD, MS
Juan Isaac, RN, BSN**
Carlos Lorenzo, MD, PhD
Arlene Martinez, RN, BSN, CDE
Monica Salazar
Tatiana Walker, RD, MS, CDE

University of Colorado (Denver, CO)

Dana Dabelea, MD, PhD*
Richard F. Hamman, MD, DrPH*
Patricia V. Nash, MS**
Sheila C. Steinke, MS**
Lisa Testaverde, MS**
